# Application of chimeric mice with human hepatocytes for the evaluation of metabolically activated genotoxic substances

**DOI:** 10.1186/s41021-026-00362-2

**Published:** 2026-06-19

**Authors:** Mayu Fujikawa, Ryoko Matsuyama, Chihiro Yamasaki, Chise Tateno, Hiroyuki Asano

**Affiliations:** 1 Advanced Research Laboratory for Fundamental Technologies, Sumitomo Chemical Co., Ltd., 1-98, Kasugadenaka 3-chome, Konohana-ku, Osaka, 554-8558 Japan; 2 PhoenixBio Co., Ltd., 3-4-1, Kagamiyama, Higashi-Hiroshima, 739-0046 Japan; 3 Graduate School of Biomedical and Health Sciences, Hiroshima University, 1-2- 3, Kasumi, Minami-ku, Hiroshima, 734-8553 Japan; 4 School of Pharmaceutical Sciences, Wakayama Medical University, 25-1, Shichibancho, Wakayama, 640-8156 Japan

**Keywords:** Liver micronucleus assay, PXB-mouse, Diethylnitrosamine, Repeated-dose liver micronucleus assay, Collagenase, Hepatocyte

## Abstract

**Background:**

The in vivo micronucleus (MN) assay is used for evaluation of chromosomal aberration induced by chemicals, and the liver MN assay can detect genotoxic compounds which require metabolic activation to induce micronucleus formation. However, species differences in liver metabolism are known, and therefore results obtained from mouse and rat liver MN assays may not always be directly extrapolated to humans. Chimeric mouse with human hepatocytes (the PXB-mouse®) is expected to be used in genotoxicity studies as an animal model of metabolic reactions in humans. To date, the use of the liver MN assay with PXB-mice has been reported, but its application has been limited to compounds that do not require metabolic activation to induce chromosomal aberrations.

**Results:**

In this study, we used the liver MN assay in PXB-mice to evaluate diethylnitrosamine (DEN) and N-nitrosodimethylamine (NDMA), both of which require metabolic activation to induce chromosomal aberrations. After repeated administration of DEN for 14 days or NDMA for 28 days, statistically significant increases in micronucleus frequency were observed.

**Conclusion:**

From the results of this examination, it was demonstrated that the liver MN assay using PXB-mice can detect the genotoxicity of compounds that require metabolic activation to exert genotoxicity. Although some issues remain, such as the presence of residual mouse hepatocytes, the liver MN assay using PXB-mice is expected to provide supportive information for more accurate evaluation of chemical genotoxicity in humans.

**Supplementary Information:**

The online version contains supplementary material available at 10.1186/s41021-026-00362-2.

## Introduction

The in vivo micronucleus (MN) assay is a genotoxicity test used to detect the potential of chemical substances to induce chromosomal aberrations, and the method of using erythrocytes from bone marrow (BM), or peripheral blood (PB) in in vivo MN assays has been adopted as an Organization for Economic Co-operation and Development guideline. However, it is known that some compounds need to be metabolically activated to cause chromosomal aberrations, and that such substances, the limited exposure of BM cells to active metabolites, or the short life-span of active metabolites generated in the liver may lead to negative results in the BM-MN assay [1]. The MN assay using hepatocytes (liver MN assay), which targets a major metabolic organ, has been utilized to solve these problems. Especially, for substances with chromosome aberration-inducing potential under conditions of metabolic activation in vitro, it has been reported that the liver MN assay can provide supporting information when bone marrow exposure level is low [2, 3].

The use of the liver MN assay, primarily in rats, has been reported in multiple studies. Micronuclei are formed when cells divide following exposure to a compound or its metabolite. Since hepatocytes replicate relatively slowly in adult animals, the liver MN assays in previous research studies were conducted in animals undergoing partial hepatectomy (PH method) [4] or in juvenile rats with active hepatocyte division (JR method) [5]. Both approaches have disadvantages, namely, in the PH method, enzymatic activities and the level of cytochrome P-450 is decreased by partial hepatectomy, with no subsequent recovery [6]. In the JR method, metabolism in juvenile animals might not be fully comparable to that in adult animals. Therefore, as an alternative to these methods, a 14- or 28-day repeated-dosing method was developed in the 2010s [7]. As recommended in the International Workshop on Genotoxicity Testing (IWGT), the international genotoxicology expert meeting [2], this method is widely used.

The toxicity profile of chemicals occasionally varies across species [8], and one reasons for this variability is differences in metabolism [9]. If the metabolites produced in rodents differ qualitatively or quantitatively or both from those in humans, the results of the liver MN assay using rodents can not be directly extrapolated to humans. The chimeric mouse humanized with human hepatocytes (PXB-mouse) is expected to serve as an evaluation system that more accurately reflects metabolic reactions in humans. It has been reported that the livers of PXB-mice possess functions similar to those of human hepatocytes in terms of mRNA and major metabolic enzyme expression levels [10, 11]. PXB-mice have also been used for the human health risk assessment of chemicals [12], in addition to predicting human pharmacokinetics [13] and searching for human-specific metabolites [14]. Although the use of liver MN assays with PXB-mice has also been reported, its use has been restricted to evaluating substances that do not require metabolic activation to induce chromosomal aberrations (N-ethyl-N-nitrosourea, ENU) [15]. In this study, we determined the applicability of the test system to genotoxic compounds requiring metabolic activation by performing the liver MN assay in PXB-mice to test two such compounds, diethylnitrosamine (DEN) and N-nitrosodimethylamine (NDMA).

## Materials and methods

### Chemicals

The study used DEN (lot no. FBMVM, purity 93.58%, Tokyo Chemical Industry Co., Ltd., Tokyo, Japan) and NDMA (lot no. PLSBI, purity 99.9%, Tokyo Chemical Industry Co., Ltd., Tokyo, Japan). These chemicals were dissolved in physiological saline (Otsuka Pharmaceutical Factory, Tokyo, Japan).

### Animals and husbandry

All experiments using animals were performed in compliance with institutional guidelines, which are in compliance with Japanese laws, and were approved by the Institutional Animal Care and Use Committees of PhoenixBio Co., Ltd. and Sumitomo Chemical Co., Ltd.

Male PXB-mice (17–18 weeks old) transplanted with human hepatocytes were prepared by PhoenixBio Co., Ltd. Human hepatocytes were obtained from a donor (Lot 195, a 2-year-old Hispanic healthy girl) by BD Biosciences (San Jose, CA). The replacement index (RI) is the percentage of human hepatocytes in the liver and is evaluated by measuring human albumin in blood [16, 17]. The RI values in the PXB-mice used in this study were determined prior to their use, and ranged from 89% to 98%. The PXB-mice were housed in a room maintained at 19–26 °C and 35%–70% relative humidity, with 10 air changes per hour and a 12-h/12-h light/dark cycle.

### Dosing

**Fig. 1:**
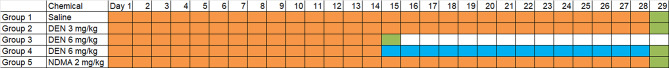
Experimental design. Orange cells show treatment days, light blue cells show withdrawal days and green cells show specimen preparing days

DEN and NDMA were dissolved in physiological saline and were orally administered. All dosing mixtures were administered at 10 mL/kg using a 1 mL syringe (Terumo, Tokyo, Japan) affixed to a sonde (Fuchigami Instruments Ltd., Kyoto, Japan). Before the MN assay, DEN was repeatedly administered to two PXB-mice at a dose of 12.5 mg/kg/day as previously reported in rats [7]. Continuous administration for 28 days was initially planned, however, on day 4, dosing was terminated due to an average body weight loss of 17% from the start of administration. Based on these results, DEN was administered at doses of 6 mg/kg/day for 14 days and 3 mg/kg/day for 28 days. The 3 mg/kg/day group was sampled on day 29 (Group 2). The 6 mg/kg/day group was divided into two groups: Group 3, sampled 1 day after the end of the treatment period (day 15), and Group 4, sampled 14 days after the end of treatment period (day 29). NDMA was administered at 2 mg/kg/day for 28 days and specimens were prepared on day 29 (Group 5), as previously reported in rats. In addition, a control group was recieved saline at 10 mL/kg for 28 days (Group 1) (Fig. 1). Group 1 consisted of two animals, whereas each of the remaining groups consisted of three animals.

### Specimen preparation

Using a slightly modified version of the previously reported method, we prepared hepatocyte specimens from the formalin-fixed liver tissues. Without prior fasting, PXB-mice were euthanized by exsanguination from the abdominal aorta under isoflurane anesthesia and their livers were collected, weighed, and fixed in neutral phosphate buffered 10% formaldehyde solution. After fixation in formaldehyde solution for more than 5 days, a small portion of the fixed liver tissue was cut into approximately 5 mm-cubes and thoroughly washed with water. The cubes were incubated in 12 M aqueous solution of potassium hydroxide (KOH; KANTO CHEMICAL CO., INC., Tokyo, Japan) at room temperature for about 18 h and then washed thoroughly with water to remove the KOH solution. The liver cubes were then mashed using a BioMasher SP homogenizer (Nippi, Inc., Tokyo, Japan), filtered through a 100 μm mesh cell strainer (Corning, Inc., USA), and suspended in water to disperse the hepatocytes. The hepatocyte suspensions were centrifuged at 80×*g* for 5 min and washed in 10% phosphate-buffered formalin. Centrifugation and washing steps were repeated 5 times or more. The pellet of hepatocytes was suspended with 10% phosphate-buffered formalin and kept in a refrigerator until ready for analysis.

### Observation

All samples were coded and analyzed in a blinded manner. Cell suspensions fixed in 10% neutral buffered formalin solution were dropped onto CREST-coated glass slides and air-dried. After washing the slides with PBS (-), we added 0.1% Triton X-100 in PBS (-) and incubated the slides for 10 min at room temperature. After washing with PBS (-) and Milli-Q, the slides were stained with DAPI and observed under a fluorescence microscope (U excitation) equipped with 20× or 40× dry objectives (15× eyepiece). Especially micronuclei were confirmed at 40×. For each animal, micronucleated cells and mitotic cells were scored in 4,000 cells in the DEN- and NDMA-treated groups and in 8,000 cells in the control group to maintain statistical power.

### Statistical analysis

Statistical analysis for the incidence of micronucleated hepatocytes was performed using the Kastenbaum-Bowman tables that were based on the binomial distribution [18]. The levels of significance were set at *p* = 0.05 and 0.01 (one-tailed). Mitotic indices (MI) were statistically analyzed by Dunnett’s test. The levels of significance were set at *p* = 0.05 and 0.01 (two-tailed). Body weights, body weight gains, and liver weights were statistically analyzed. The F-test and *t*-test were applied to determine the statistical significant differences between the control group and the treated groups. If *p* > 0.05 in the F-test, the Student's *t*-test was used, and if *p* < 0.05 in the F-test, the Welch's *t*-test was used. The levels of significance were set at *p* = 0.05 and 0.01 (two-tailed).

## Results

### Clinical observations

In the control group, one out of three animals died on day 17 for unknown reasons. In the other groups, including the treatment groups, no deaths or abnormal signs due to administration were observed (Supplementary Table S1).

### Body weight

**Fig. 2:**
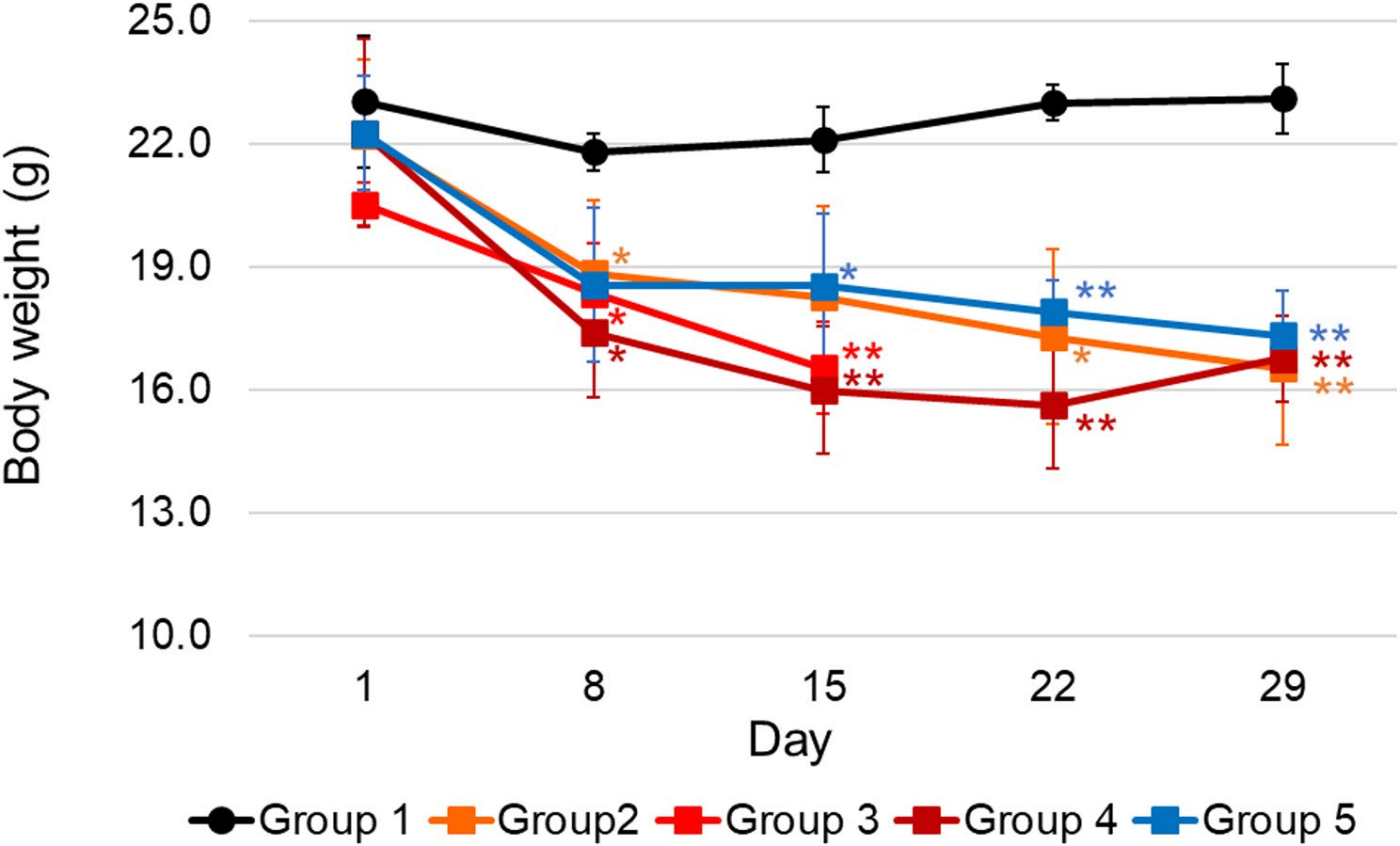
Body weight changes of PXB-mice in each group. Values are presented as the mean and SD. * *p* < 0.05, ** *p* < 0.01

The body weight of the control group did not change significantly during the treatment period. All treatment groups showed a statistically significant decrease in body weight compared with the control group (Fig. 2 and Supplementary Table S3). Receiving 6 mg/kg of DEN for 14 days, Groups 3 and 4 experienced a statistically significant decrease from the control group’s body weight throughout almost the whole treatment period. In Group 4, body weight recovery did not occur immediately after the completion of the treatment period, with the reduction reaching a maximum of 7.3 g (31.5%, day 19) compared with the control group’s body weight. At 29 days, body weight recovered slightly, but was 6.3 g (27.4%) less than in the control group. In Groups 2 (DEN 3 mg/kg/day for 28 days) and 5 (NDMA 2 mg/kg/day for 28 days), body weights decreased throughout the treatment period. In Group 2, we observed statistically significant decreases compared with those in the control group sporadically until day 17 and consistently from day 18 onward. The difference from the control group’s body weight reached its maximum on day 29, with a value of -6.57 g. In Group 5, significant decreases were observed almost consistently from day 15 onward. The difference from the control group’s body weight also peaked on day 29, with a value of -5.77 g.

### Frequency of micronucleus and mitotic cells in liver

**Fig. 3:**
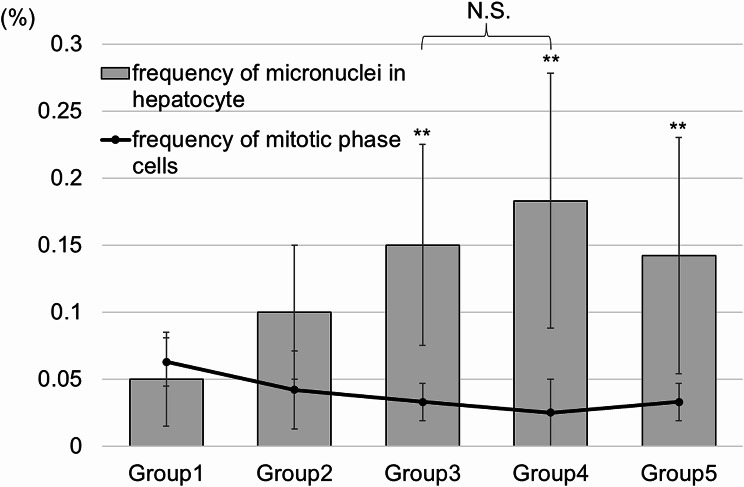
The results of the liver micronucleus assay with PXB-mice. Values are presented as the mean and SD. Differences in the incidences of micronucleated hepatocytes between the control (Group 1) and the test chemical treated groups, as well as between Group 3 and Group 4, were analyzed by Kastenbaum and Bowman test. **: *p* < 0.01, N.S.: not significant. Differences in the incidences of mitotic phase cells between the control (Group 1) and test chemical treated groups were analyzed by Dunnett’s multiple comparison test

In Groups 3 and 4, which received 6 mg/kg/day of DEN, micronucleus frequency in the liver was significantly increased compared to the control group (Fig. 3). There was no statistical difference in micronucleus frequency between Groups 3 and 4, with or without a withdrawal period. A significant increase in micronucleus frequency was also observed in Group 5, which received NDMA compared to the control group. In Group 2 where DEN was administered at a dose of 3 mg/kg/day, there was no statistically significant increase in micronucleus frequency compared with that in the control group. For MI, no statistically significant difference was observed between the control group and each treated group. Human hepatocyte RI in PXB-mice was high (> 89%) for all animals, and no correspondence was found between individual values of RI and micronucleus frequency or values of MI in the liver in each group (Table 1).

### Liver weight

Groups 2, 4, and 5 showed statistically significant decreases in absolute liver weight on Day 29, but no significant differences in relative liver weight (Table 2, Supplementary Table S2)

**Table 1:** The individual values of liver micronucleus frequency, MI and RI

Group	Chemical	Dose (mg/kg/day)	Days of dosing	Day of sample preparation	RI (%)	MI (%)	Frequency of micronuclei(%)
1	Control	-	28	29	94	0.075	0.075
					89	0.050	0.025
2	DEN	3	28	29	90	0.025	0.100
					93	0.025	0.050
					94	0.075	0.150
3	DEN	6	14	15	94	0.025	0.075
					95	0.050	0.150
					91	0.025	0.225
4	DEN	6	14	29	95	0.025	0.075
					95	0.000	0.225
					91	0.050	0.250
5	NDMA	2	28	29	90	0.050	0.050
					94	0.025	0.150
					98	0.025	0.225

**Table 2:** Mean of liver weight and Relative value of liver weight to body weight

	Day of Measurement	Liver weight		Relative value of liver weight to b.w.	
		Mean	SD	Mean	SD
Group 1	29	3.6	0.14	0.156	0.0119
Group2	29	2.6*	0.32	0.156	0.0175
Group4	29	2.7*	0.32	0.159	0.0131
Group5	29	2.5*	0.38	0.142	0.0144
Group3	15	2.2	0.70	0.132	0.0340

Differences in the liver weight and the relative value of liver weight to body weight (b.w.) between the control group and Group 2, 4 and 5, were analyzed by t-test. *: *p* < 0.05

## Discussion

The PXB-mouse is expected to increase the precision of assessment of the effects of chemicals on human health. The aim of the present study was to confirm whether the MN assay with PXB-mice can be applied to compounds that exhibit genotoxicity after metabolic activation. The liver MN assay using PXB-mice was performed to evaluate the genotoxicity of DEN and NDMA, both of which are genotoxic when metabolically activated. Administering them continuously for 14 or 28 days as recommended for non-chimeric animals led to a statistically significant increase in liver micronuclei in both the DEN- and NDMA-treated groups compared to the control group. It has been reported that CYP2E1 (cytochrome P450 2E1) is involved in the metabolic activation of both DEN and NDMA [19]. Although the hepatocyte donor used in the present study was 2 years old, a previous study reported that in PXB-mice transplanted with hepatocytes from 6-month-old and 6-year-old donors, the expression levels of CYPs, including CYP2E1, were comparable to those in adult human hepatocytes (from 25, 28, 57, 61 years old) (GEO data set available at https://www.ncbi.nlm.nih.gov/sites/GDSbrowser?acc=GDS4327) [10]. These findings suggest that donor age does not markedly affect CYP expression in PXB-mice. The present results are consistent with these characteristics of PXB-mice and provide evidence supporting their usefulness for genotoxicity evaluation. However, since both compounds used in this study are metabolized by CYP2E1, further investigation is necessary to determine whether this test system can be applied to compounds that are metabolized by other metabolic enzymes and exhibit genotoxicity.

For precise evaluation of genotoxicity in humans, it is important to use animals with a high human hepatocyte RI. The RI values of the PXB-mice used in this study were all approximately 90% at the time of administration (18 weeks of age). PXB-mice are generated by transplanting human hepatocytes into immunodeficient mice with liver injury [16, 17]. After transplantation, the RI increases by virtue of human hepatocytes actively proliferating in the mouse liver. It has been reported that the RI is well correlated with the concentration of human albumin (h-alb) in the blood of PXB-mice, and the h-alb level increases rapidly in younger animals, and then rises more gradually as the animals age. The rate of increase in h-alb becomes more gradual after 15 weeks of age, however, a steady increase is observed up to 29 weeks of age [17]. Micronuclei are formed when cells divide after exposure to compounds or their metabolites. Therefore, there was concern that the sensitivity of genotoxicity detection will decrease after 15 weeks of age when human hepatocytes proliferate at a slower rate. However, the increase in liver micronucleus frequency in animals administered DEN or NDMA demonstrated that compounds exhibiting genotoxicity via metabolic activation can be detected by the liver MN assay using PXB-mice. In addition, although the PXB-mice used in this study showed some variation in RI at 18 weeks of age (ranging from 89 to 98), no correlation was observed between RI values and liver micronucleus frequency within each dosing group. This suggests that cell division frequency at 18 weeks does not depend on the RI value. On the other hand, even after 18 weeks of age, it is estimated that approximately 10% of mouse hepatocytes remain. Further investigation is necessary to clarify the impact of the residual mouse hepatocytes on the test results.

Furthermore, regarding hepatocyte proliferation, it has been reported that hepatocyte division can be suppressed by factors such as reduced food intake and weight loss [20]. In this study, because decreased body weight and lower liver weights were observed in the compound-treated groups, there was concerned that suppression of hepatocyte division may have occurred. However, no statistically significant decrease in MI values was observed in the DEN- and NDMA-treated groups compared with the control group. In addition, because the MI values in Groups 3 and 4, which received DEN at 6 mg/kg/day for 14 days, were comparable to the MI values reported for rats administered DEN at 6.25 mg/kg/day for 14 days [21], marked suppression of hepatocyte proliferation was considered unlikely to have occurred in PXB-mice. Nevertheless, the frequency of micronucleated hepatocyte was lower in this study (< 0.2%) than that reported in rats (approximately 0.6–0.8%) [21]. Although our results demonstrate that compounds showing clear positive responses, such as DEN and NDMA used in the present study, can be detected, it is considered necessary to confirm the sensitivity of this test system using compounds with a weak potential to induce chromosomal aberrations.

Regarding the dose levels of compounds, while NDMA could be administered to PXB-mice for 28 days at the same dose as in non-chimeric animals, the maximum tolerated dose of DEN for 14 or 28 days of administration was significantly lower in PXB-mice than in rats or mice. In rats or mice, it has been reported that DEN can be administered at 12.5 mg/kg/day for 14 or 15 days [7]. In contrast, when PXB-mice were administered 12.5 mg/kg/day of DEN, a significant decrease in body weight was observed by day 4, making it difficult to continue dosing. Therefore, 6 mg/kg/day was used in this study. When evaluating compounds with unknown genotoxicity, if the maximum tolerated dose is significantly lower in chimeric animals compared with non-chimeric animals, it is important to justify the selected dose levels, especially when the test results are negative. This justification can be made by comparing the dose levels with the estimated human exposure concentrations.

In the present study, a repeated-dose liver MN assay using PXB-mice with approximately 90% of RI was performed for compounds requiring metabolic activation to exert genotoxicity. The genotoxicity of these compounds was successfully detected. PXB-mice with human hepatocytes are expected to yield a more precise assessment of human health risks by enabling the evaluation of the genotoxicity of human metabolites. Although further experiments are required to confirm the effect of residual mouse hepatocytes and the sensitivity of the test system, it is considered that the liver MN assay using PXB-mice will provide valuable insights into the assessment of the genotoxicity of chemicals in humans.

## Supplementary Information

Below is the link to the electronic supplementary material.

Supplementary Material 1

## Data Availability

The datasets used during the current study are available upon reasonable request.
