# Supplementary material for: Application of chimeric mice with human hepatocytes for the evaluation of metabolically activated genotoxic substances
Source: Genes Environ. 2026 Jun 19;48:15. doi: 10.1186/s41021-026-00362-2 (PMC13435476; doi:10.1186/s41021-026-00362-2)
Supplement: Supplementary file 1 [file 41021_2026_362_MOESM1_ESM.docx]

Supplementary Table S1. The results of clinical observation.

|  | Day 1-7 | Day 8-14 | Day 15-21 | Day 22-29 |
| --- | --- | --- | --- | --- |
| Group 1 | none | none | none | none |
| Group 2 | none | none | none | none |
| Group 3 | none | none | none | none |
| Group 4 | Loss of hair on the nose (2/3) | Loss of hair on the nose (1/3) | none | none |
| Group 5 | Loss of hair on the nose (1/3) | Loss of hair on the nose (1/3) | Loss of hair on the nose and the back (1/3) | Loss of hair on the nose and the back (1/3) |

Supplementary Table S2. Individual values of body weight and liver weight on the day of preparation.

| Group | Chemical | Dose (mg/kg/day) | Treatment period (day) | Sample preparation (day) | Body weight (g) | Liver weight (g) |
| --- | --- | --- | --- | --- | --- | --- |
| 1 | Saline | 10mL/kg | 28 | 29 | 22.5 | 3.7 |
|  |  |  |  |  | 23.7 | 3.5 |
| 2 | DEN | 3 | 28 | 29 | 18.7 | 2.8 |
|  |  |  |  |  | 15.5 | 2.2 |
|  |  |  |  |  | 15.4 | 2.7 |
| 3 | DEN | 6 | 14 | 15 | 17.8 | 2.9 |
|  |  |  |  |  | 15.7 | 1.5 |
|  |  |  |  |  | 16.1 | 2.2 |
| 4 | DEN | 6 | 14 | 29 | 17.8 | 2.8 |
|  |  |  |  |  | 16.8 | 2.9 |
|  |  |  |  |  | 15.7 | 2.3 |
| 5 | NDMA | 2 | 28 | 29 | 17.2 | 2.2 |
|  |  |  |  |  | 16.3 | 2.3 |
|  |  |  |  |  | 18.5 | 2.9 |

Supplementary Table S3. Average body weight of PXB-mice; yellow cells: p<0.05, red cells: p<0.01.
